# Non-HDL-cholesterol to HDL-cholesterol ratio is an independent risk factor for liver function tests abnormalities in geriatric population

**DOI:** 10.1186/s12944-018-0940-0

**Published:** 2018-12-28

**Authors:** Tianhui An, Yi Song, Yi Yang, Mengyuan Guo, Hui Liu, Kun Liu, Zhaohui Wang

**Affiliations:** 1 Department of Geriatrics, Union Hospital, Tongji Medical College, Huazhong University of Science and Technology, Wuhan, 430022 China; 2 Department of Geriatrics, Tongji Hospital, Tongji Medical College, Huazhong University of Science and Technology, Wuhan, 430030 China; 3 Department of Cardiology, Union Hospital, Tongji Medical College, Huazhong University of Science and Technology, Wuhan, 430022 China

**Keywords:** Non-HDL cholesterol, HDL-cholesterol, Liver function tests abnormalities, Geriatric population, Risk factor

## Abstract

**Background:**

Excessive lipid depositing in liver cells could induce pathophysiological development of liver. Our study aimed to assess whether non-HDL cholesterol to HDL-cholesterol ratio (NonHDLc/HDLc) is an independent risk factor for liver function tests (LFTs) abnormalities in geriatric population.

**Methods:**

We enrolled 1745 eligible subjects (714 males, 1031 females) with normal liver function tests at baseline who participated in annual health checkup for liver disease in 2015. Logistic regression models were used to examine the independent relationship between NonHDLc/HDLc ratio and LFTs abnormalities.

**Results:**

After one year follow-up, there were 6.1% (*n* = 107) participants developed new-onset LFTs abnormalities in 2016. Equally dividing participants into tertiles according to their baseline NonHDLc/HDLc ratio levels, we found compared with tertile 1, the multivariable-adjusted ORs (95% CIs) for new-onset LFTs abnormalities of tertile 3 were 2.85 (1.18–6.93), *P* = 0.021. In stratified analysis, compared with controls, the correlation between NonHDLc/HDLc ratio and incidence of LFTs abnormalities was more remarkable in female individuals, BMI > 24 individuals and free of diabetes individuals.

**Conclusion:**

Our study suggests that NonHDLc/HDLc ratio is an independent risk factor for LFTs abnormalities in geriatric population, and assessment of NonHDLc/HDLc ratio may help early identify high risk people of liver diseases.

**Trial registration:**

Trial registration in the Ethics Committee of Tongji Medical College, Huazhong University of Science and Technology (IORG No: IORG0003571). Registered 3 March 2015.

**Electronic supplementary material:**

The online version of this article (10.1186/s12944-018-0940-0) contains supplementary material, which is available to authorized users.

## Introduction

Liver disease usually accompanied by LFTs abnormalities is a significant health burden worldwide, especially among western countries and China [1, 2]. Apart from traditional major risk factors as hepatic virus and alcoholic abuse, dyslipidemia can also be predisposed to the pathophysiological development of liver and further cause LFTs abnormalities [3, 4].

Dyslipidemic profile caused by obesity or insulin resistance is characterized by an increased large amount of VLDL, small dense LDL, IDL, and a decreased HDL which correlates with intrahepatic lipid content [5–7]. Furthermore, excessive lipid depositing in liver cells induce complex inflammation states including oxidative stress, cytochrome P450 activation, lipid peroxidation, increased inflammatory cytokine production such as TNF-α, TGF-β and ILs, and all the above would lead to elevated aminotransferase and other LFTs abnormalities [8].

Non-HDL-cholesterol (NonHDLc), which roughly equals the total amount of LDL, VLDL, IDL and lipoprotein(a), is highlighted as a key secondary target of lipid lowering [9, 10]. With respect to NonHDLc/HDLc ratio, the UK Prospective Diabetes Study found NonHDLc/HDLc ratio to be better than nonHDL-C as a predictor of coronary heart disease (CHD) in type 2 diabetes patients [11, 12]. More importantly, this ratio has a stronger association with CHD incidence in chronic kidney disease (CKD) patients compared with ApoB/ApoA1 [13]. Additionally, NonHDLc/HDLc ratio is an ideal marker in identifying metabolic syndrome and insulin resistance, which are two risk factors for both liver diseases and CHD [14].

Considering similar pathogenesis between liver disease and CHD—obesity, insulin resistance, metabolic syndrome, inflammation and so on— we are interested in exploring whether NonHDLc/HDLc ratio is also an independent risk factor for liver disease charactered by LFTs abnormalities in geriatric population as of CHD.

Herein we designed this study to investigate the correlation between NonHDLc/HDLc ratio and newly-presented abnormal LFTs, so as to explore whether this ratio is an independent risk factor for LFTs abnormalities in geriatric population.

## Materials and methods

### Ethic statement

All study was in accordance with relevant guidelines of Medical Ethics Committee and approved by the Institutional Ethics Committee of Tongji Medical College of Huazhong University of Science and Technology (HUST).

### Study participants

**Fig. 1:**
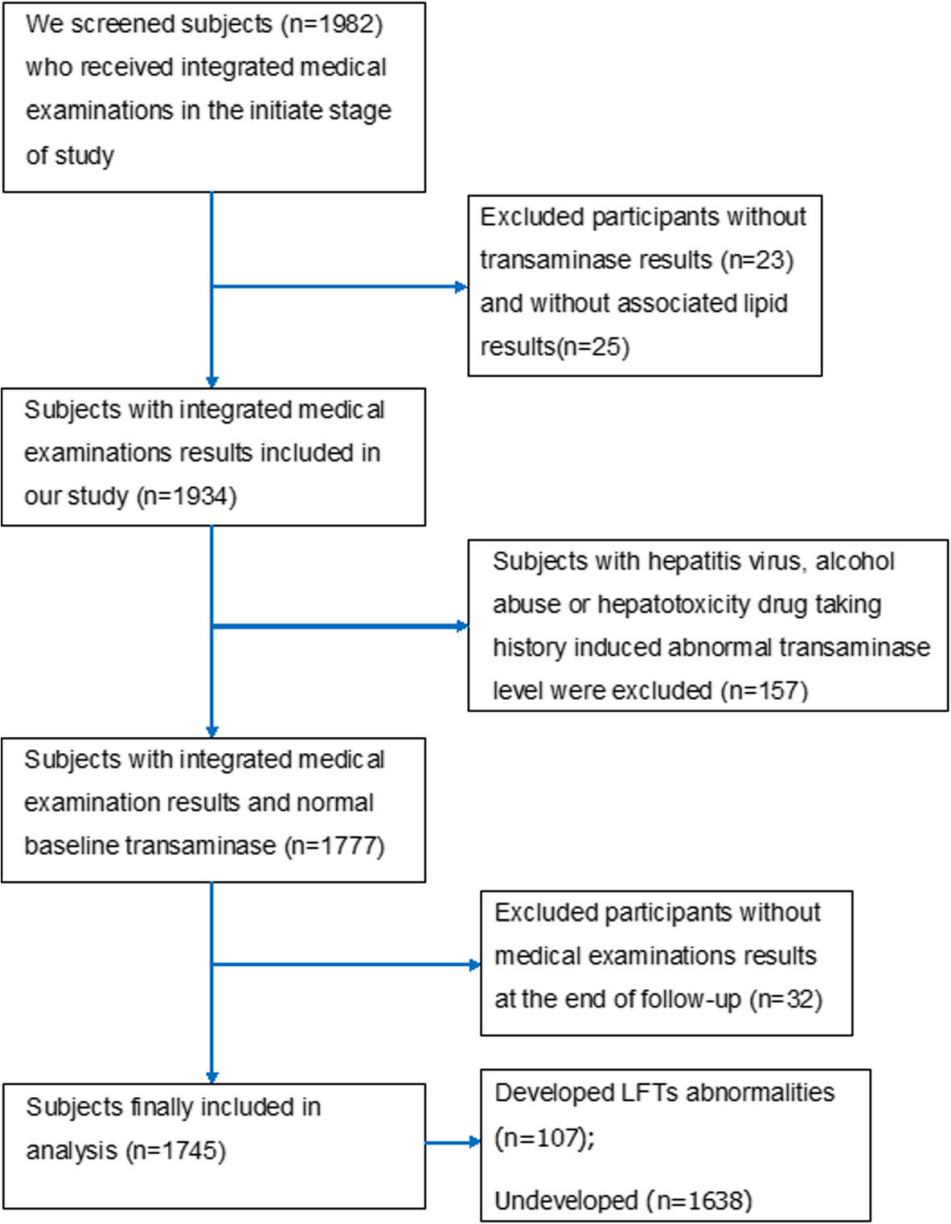
Flowchart of our prospective cohort study. Numbers of potential participants, reasons for exclusion and the final subjected included in analysis

The study subjects and data were derived from community-dwelling residents who had undergone annual medical examinations in Shui Guohu Hospital from 2015 to 2016. In the initiate stage of study, 1982 subjects received integrated medical examinations and were followed for 1 year, and the corresponding data of two years was collected in the questionnaires or in our electronic medical record system. 23 participants without transaminase results and 25 participants without associated lipid results were excluded. Additionally, after checking patients’ specific ID number to access their examination data, we excluded 157 individuals with abnormal transaminase level induced by hepatitis virus, alcohol abuse or hepatotoxicity drug taking. Then 32 participants whose associated data missed at the end of the follow-up were excluded. Finally, the eligible sample size for analyses was 1745 in the present study. Flowchart of the patient screening process is depicted in Fig. 1.

### Biochemical measurements and outcomes

Standardized physical examination procedures were performed. Sociodemographic information and medical history were collected through physical examination questionnaires by trained doctors, including age, sex, marital status, education status, smoking status, alcohol consumption, physical activity, drug taking(especially medications with known hepatotoxicity), history of diabetes, hypertension and liver disease. Then, participants were measured for anthropometric data containing height, weight, systolic blood pressure and diastolic blood pressure. Height and weight data were collected when subjects wore light clothes and were barefoot. We calculate body mass index (BMI) as weight in kilograms divided by the square of the height in meters. Blood pressure was measured in the participants’ right arm using a standard mercury sphygmomanometer with subjects in a seated position after a rest of at least 10 min. The mean values of the three readings were used for analysis.

Additionally, the hospital laboratory measured biochemical markers including fasting plasma glucose, total cholesterol, Triglyceride, low-density (LDL) lipoprotein, high-density lipoprotein (HDL), total bilirubin, uric acid, alanine aminotransferase (ALT), aspartate aminotransferase (AST). All blood samples were obtained after an overnight fasting, and were processed within two hours of collection.

### Diagnostic criteria

liver function tests abnormalities were defined as ALT no less than 35 U/L or AST no less than 40 U/L complying with Chinese standard of diagnosis. In this paper, we identified newly appearance LFTs abnormalities by identifying participants whose baseline data (year 2015) of ALT and AST were normal but follow-up data (year 2016) were higher than diagnostic criteria.

NAFLD was defined as the presence of fatty liver disease (FLD) based on abdominal B-type ultrasound inspection after excluding excessive significant alcohol consumption, medications with known hepatotoxicity and other liver disease including hepatitis virus infection, chronic hepatitis, hepatic cirrhosis or carcinoma.

### Statistical analysis

All statistical analyses were performed with SPSS version 17. Continuous variables were presented as mean ± SD, and categorical variables were expressed as percentages (%). The differences between groups were analyzed using one-way ANOVA for normally distributed continuous variables and the chi-squared test for categorical variables. Univariate and multivariable logistic regression models were used to calculate odds ratios (OR) and 95% confidence intervals (CI) for the associations of NonHDL/ HDL ratio and LFTs abnormalities. Model 1 adjusted for age, gender, BMI, systolic BP, diastolic BP, triglycerides, total cholesterol, HDL-cholesterol, Non-HDL-cholesterol, fasting plasma glucose; Model 2 adjusted for Model 1 plus serum creatinine, blood urea nitrogen, alanine transaminase, aspartate aminotransferase, total bilirubin, history of hypertension and history of diabetes mellitus. Two tailed *P*-values were reported for all analyses and *P*-value< 0.05 was considered to be statistically significant.

## Results

### Baseline characteristics

A total of 1745 eligible subjects (714 males, 1031 females) were enrolled in the study. Dividing the participants into tertiles according to NonHDLc/HDLc ratio, we compared the baseline characteristics of them in Table 1. The subjects with higher NonHDLc/HDLc level at baseline were more likely to have lower HDL-cholesterol and higher BMI, diastolic BP, triglycerides, total cholesterol, LDL-cholesterol, non-HDL-cholesterol, fasting glucose, serum creatinine and ALT. For example, ALT (from 15.9 ± 0.3, 16.7 ± 0.3 to 17.2 ± 0.3, *p* = 0.003) increased as tertile arose. Conversely, AST (from 21.9 ± 0.2, 21.3 ± 0.2 to 20.9 ± 0.2, *p* = 0.001) decreased as the tertile arose and another LFTs index T-Bil (from 12.3 ± 7.4, 11.9 ± 6.5 to 11.5 ± 4.1, *p* = 0.063) had no significant difference among three tertiles. Table 2 then compared the level of ALT, AST and T-Bil at the end of following-up and found that ALT(from 17.8 ± 0.6, 18.6 ± 0.5 to 20.0 ± 0.8, *p* = 0.047) persisted significantly as the tertile increased but AST (from 23.2 ± 0.5, 23.0 ± 0.4 to 23.2 ± 0.5, *p* = 0.950) and T-Bil (from 11.6 ± 0.2, 11.7 ± 0.2 to 11.2 ± 0.2, *p* = 0.132) almost had no difference in tertiles. These results suggested that people with higher NonHDL/HDL ratio are more inclined to have higher ALT, and this ratio could significantly increase AST and minor increase TBil in participants.

**Table 1:** Clinical and biochemical characteristics by tertiles of Non-HDL/HDL levels

Variables	Non-HDL / HDL			*P*-value
	Tertile 1	Tertile 2	Tertile 3	
Sample size	582	582	581	
Non-HDL / HDL	≤2.23	2.23 —2.96	≥2.96	
Age (years)	71.5 ± 5.9	70.2 ± 5.3	70.0 ± 5.2	< 0.001
Sex(Femal/Male)	346/236	358/224	327/254	0.718
BMI (kg/m^2^)	22.8 ± 3.2	23.5 ± 2.9	23.9 ± 2.9	< 0.001
Systolic BP (mmHg)	128.6 ± 16.7	129.9 ± 16.1	130.6 ± 15.4	0.086
Diastolic BP (mmHg)	76.0 ± 9.6	78.2 ± 9.6	78.9 ± 9.6	< 0.001
Triglycerides (mmol/L)	1.0 ± 0.4	1.4 ± 0.6	1.9 ± 1.0	< 0.001
Total cholesterol (mmol/L)	4.1 ± 0.8	4.5 ± 0.8	4.9 ± 1.0	< 0.001
LDL- cholesterol (mmol/L)	2.8 ± 0.8	3.4 ± 0.9	3.6 ± 1.1	< 0.001
HDL-cholesterol (mmol/L)	1.5 ± 0.4	1.3 ± 0.2	1.1 ± 0.2	< 0.001
Non-HDL-cholesterol (mmol/L)	2.6 ± 0.6	3.3 ± 0.6	3.8 ± 0.8	< 0.001
Fasting glucose (mmol/L)	5.2 ± 1.1	5.4 ± 1.6	5.6 ± 1.5	< 0.001
Serum creatinine (μmol/L)	70.3 ± 18.9	71.1 ± 33.9	74.1 ± 25.6	0.044
Blood urea nitrogen (mmol/L)	5.8 ± 1.5	5.8 ± 4.4	5.7 ± 2.4	0.909
Alanine Transaminase(U/L)	15.9 ± 6.3	16.7 ± 6.3	17.2 ± 6.3	0.003
Aspartate Transaminase(U/L)	21.9 ± 5.0	21.3 ± 4.7	20.9 ± 4.9	0.001
Total bilirubin (U/L)	12.3 ± 7.4	11.9 ± 6.5	11.5 ± 4.16.5	0.063
Hypertension, %	48.1/51.9	49.0/51.0	50.8/49.2	0.651
Diabetes mellitus, %	14.3/85.7	15.5/84.5	17.0/83.0	0.424

*BMI* body mass index, *HDL* high-density lipoprotein, *LDL* low-density lipoprotein. Data are presented as the mean ± SD. *P* value compared with tertile 1

The cut of values of Non-HDL/HDL ratio were < =2.23, 2.23–2.96, and > =2.96. *BMI* body mass index, *HDL* high-density lipoprotein, *LDL* low-density lipoprotein. Data are presented as the mean ± SD. *P* value compared with tertile 1

**Table 2:** Transaminase and total bilirubin levels in tertiles at the end of follow-up

	Tertile 1	Tertile 2	Tertile 3	*P* value
ALT	17.8 ± 0.6	18.6 ± 0.5	20.0 ± 0.8	0.047
AST	23.2 ± 0.5	23.0 ± 0.4	23.2 ± 0.5	0.950
T-Bil	11.6 ± 0.2	11.7 ± 0.2	11.2 ± 0.2	0.132

*ALT* alanine transaminase, *AST* aspartate transaminase, *T-Bil* total bilirubin

Data are presented as the mean ± SD

### Association between NonHDLc/HDLc ratio and the incidence of LFTs abnormalities in geriatric population

**Fig. 2:**
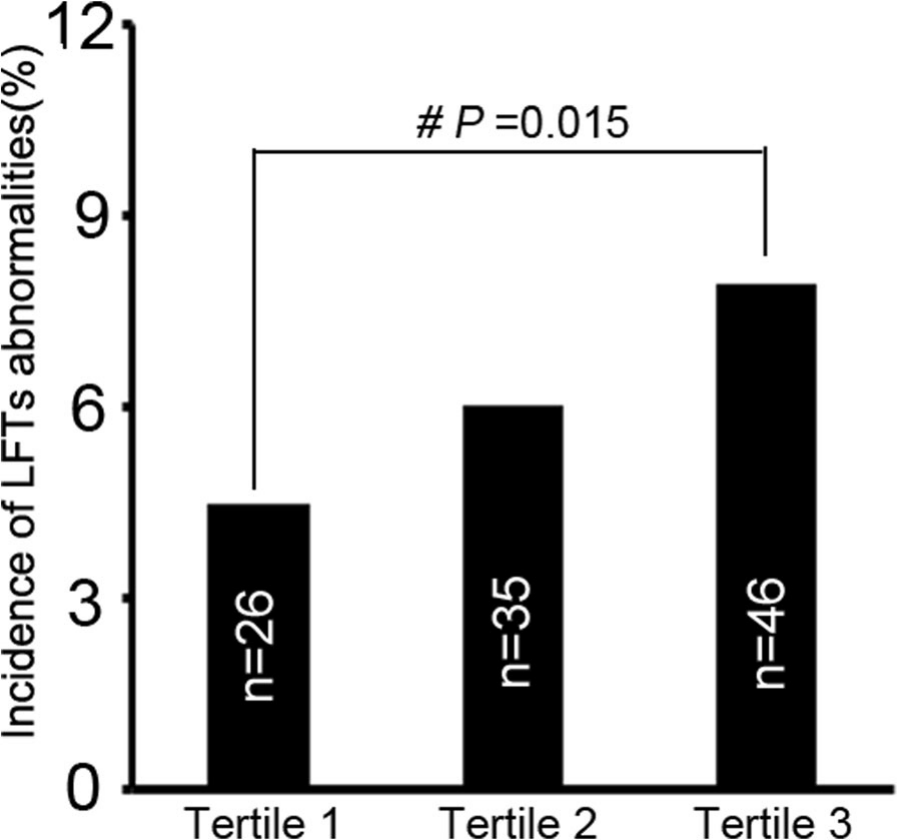
The incidence rates of LFTs abnormalities according to Non-HDL/HDL levels tertiles. The cut of values of Non-HDL/HDL ratio were < =2.23, 2.23–2.96, and > =2.96.*#P* value compared with tertile 1, *#P* < 0.05

Figure 2 presented the incidence of LFTs abnormalities according to tertiles of the NonHDLc/HDLc ratio levels among participants. The proportion who had new-onset hepatocellular injury at the end of follow-up in tertile 3 (7.9%, *n* = 46) was significantly higher than that of tertil1 (4.5%, *n* = 26) and tertile 2 (6.0%, *n* = 35), (*p* < 0.05).

As shown in Table 3, the OR (odds ratio, OR) for newly occurrence of abnormal ALT+AST (means abnormal ALT, AST or both) and ALT increased as the NonHDLc/HDLc ratio arose. Compared with tertile 1, the OR and 95% CI for tertile 3 (1.84 (1.12–3.01), *p* = 0.016 for ALT+AST; 1.81(1.08–3.03), *p* = 0.024 for ALT) were significantly higher. After being adjusted for the confounding variables, the OR and 95% CI for ALT+AST and ALT was remained significant in model 1 (2.80(1.17–6.70), *p* = 0.021 and 2.82 (1.14–7.00), *p* = 0.025, respectively). Further adjustment of serum creatinine, blood urea nitrogen, alanine transaminase, aspartate transaminase, total bilirubin, history of hypertension, history of diabetes mellitus did not affect the association, as in model 2 showed (tertile3 vs 1: OR, 2.85; 95%CI, 1.18–6.93; *p* = 0.020 for ALT+AST and OR,2.96; 95%CI, 1.17–7.48; *p* = 0.022 for ALT). However, there was no significant difference in newly occurrence abnormal AST in both models. Owing to the total number of T-Bil abnormity is relatively small, we did not include it in the end event analysis.

**Table 3:** Odds ratios (95% confidence intervals) for incident LFTs abnormalities by Non-HDL/HDL levels tertiles

	Unadjusted Odds ratio (95% CI)	*P* Value	Model 1^a^ Odds ratio (95% CI)	*P* Value	Model 2^b^ Odds ratio (95% CI)	*P* Value
ALT + AST						
NonHDLc/HDLc	1.36 (1.06, 1.73)	0.015	1.65 (1.08, 2.52)	0.020	1.67 (1.08, 2.56)	0.020
Tertiles NonHDLc/HDLc						
Tertile 1	1		1		1	
Tertile 2	1.37 (0.81, 2.30)	0.238	1.80 (0.91, 3.56)	0.091	1.80 (0.90, 3.58)	0.095
Tertile 3	1.84 (1.12, 3.01)	0.016	2.80 (1.17, 6.70)	0.021	2.85 (1.18, 6.93)	0.020
ALT						
NonHDLc/HDLc	1.35 (1.04, 1.74)	0.022	1.66 (1.07, 2.57)	0.024	1.70 (1.08, 2.66)	0.021
Tertiles NonHDLc/HDLc						
Tertile 1	1		1		1	
Tertile 2	1.35 (0.79, 2.33)	0.275	1.80 (0.89, 3.64)	0.105	1.83 (0.89, 3.78)	0.100
Tertile 3	1.81 (1.08, 3.03)	0.024	2.82 (1.14, 7.00)	0.025	2.96 (1.17, 7.48)	0.022
AST						
NonHDLc/HDLc	1.29 (0.93,1.80)	0.135	1.52 (0.85, 2.71)	0.161	1.48 (0.83, 2.63)	0.184
Tertiles NonHDLc/HDLc						
Tertile 1	1		1		1	
Tertile 2	1.37 (0.68, 2.76)	0.379	1.72 (0.69, 4.31)	0.247	1.62 (0.65, 4.04)	0.300
Tertile 3	1.67 (0.85, 3.28)	0.135	2.40 (0.72, 8.00)	0.154	2.25 (0.69, 7.38)	0.180

*HDLc* high-density lipoprotein cholesterol

^a^ Model 1 adjusted for age, gender, body mass index, systolic BP, diastolic BP, triglycerides, total cholesterol,

LDL- cholesterol, HDL-cholesterol, Non-HDL-cholesterol, fasting plasma glucose

^b^ Model 2 adjusted for model 1 plus serum creatinine, blood urea nitrogen, alanine transaminase, aspartate, transaminase, total bilirubin, history of hypertension, history of diabetes mellitus

### Stratified analysis according to gender, BMI and diabetes

Analyses results stratified by gender, BMI and diabetes were described in Table 4. The correlation between NonHDLc/HDLc ratio and incidence of LFTs abnormalities was more remarkable in female individuals (tertile3 vs 1, OR 3.50; 95% CI, 0.99–12.34; *p* = 0.046) compared with male (tertile3 vs 1, OR 2.48; 95% CI, 0.66–9.34; *p* = 0.189). Similarity, BMI > 24 individuals (tertile3 vs 1, OR 7.14; 95% CI, 1.75–29.1; *p* = 0.006) and non-diabetes individuals (tertile3 vs 1, OR 2.94; 95% CI, 1.10–7.82; *p* = 0.036) were more remarkable than BMI ≤ 24 individuals (tertile3 vs 1, OR 1.57; 95% CI, 0.41–6.00; *p* = 0.527) and Diabetes individuals (tertile3 vs 1, OR 2.08; 95% CI, 0.10–44.9; *p* = 0.505), respectively. Difference between every pair of stratified index were not statistically significant (*p* for interaction > 0.05).

**Table 4:** Multivariable-adjusted Odds ratios (95% CIs) for LFTs abnormalities based on Non-HDL/ HDL levels tertiles, stratified by sex, BMI and Diabetes respectively

	No. of events	Non-HDL / HDL			*P* value	*P* value for interaction
		ertile 1	Tertile 2	Tertile 3		
Gender						
Female	1031	1	1.94 (0.71, 5.28)	3.50 (0.99, 12.34)	0.046	0.167
Male	714	1	1.78 (0.66, 4.77)	2.48 (0.66, 9.34)	0.189	
BMI						
> 24	657	1	3.39(1.07, 0.81)	7.14 (1.75, 29.1)	0.006	0.142
< =24	1088	1	1.37 (0.52, 3.58)	1.57 (0.41, 6.00)	0.527	
Diabetes						
No	1473	1	2.00 (0.95, 4.23)	2.94 (1.10, 7.82)	0.036	0.362
Yes	272	1	0.85 (0.08, 8.91)	2.08 (0.10, 44.9)	0.505	

*BMI* body mass index

The ORs (95% CIs) were presented compared with the tertile 1, afer adjustment for underlying confounders including age, gender, body mass index, systolic BP, diastolic BP, triglycerides, total cholesterol, LDL- cholesterol, HDL-cholesterol, Non- HDL-cholesterol, fasting plasma glucose, serum creatinine, blood urea nitrogen, alanine transaminase, aspartate transaminase, total bilirubin, history of hypertension, history of diabetes mellitus

Additional file 1 Figure S1 showed the portion of LFTs abnormalities accompanied with NAFLD (non-alcoholic fatty liver disease, NAFLD) at the end of follow-up significantly increased (from 17.2 ± 5.4‰, 34.4 ± 7.6‰ to 53.4 ± 7.6‰, *p* = 0.004) as tertile increased . Same trends existed in the portion of NAFLD (from 33.5 ± 2.0%, 42.3 ± 2.1% to 50.9 ± 2.1%, *p* < 0.001) as presented in Additional file 2: Figure S2. Because there is no baseline data of liver ultrasound, we haven’t analysed whether NonHDLc/HDLc is an independent risk factors of NAFLD.

## Discussion

In this prospective cohort study, we provided evidence for the first time that NonHDLc/HDLc ratio is an independent risk factor for LFTs abnormalities in geriatric population. More importantly, it persisted significantly after adjustment for classical risk factors including hypertension, diabetes, lipid markers, age, gender, BMI and so on. After performing stratified analysis, compared with controls, the correlation between NonHDLc/HDLc ratio and incidence of LFTs abnormalities was more remarkable in female individuals, BMI > 24 individuals and free of diabetes individuals.

In addition, portion of NAFLD patients also increased as NonHDLc/HDLc tertile arose.

Previous studies have demonstrated the correlation between dyslipidemia and liver disease. For example, obesity or dyslipidemia is one of the primary risk factors of NAFLD, which is the leading cause of chronic liver disease in western world [15]. Many clinical trails showed that lipid-lowing drug statin treatment could substantially improve liver test indexes of patients [16, 17].

The pathological process of dyslipidemia hinted that excessive cellular lipid accumulation occurred not only in adipose tissue but also in organs such as liver. Ectopic lipid overloading in hepatocytes generate reactive oxygen species resulting in lipid peroxidation, oxidative stress state and the releasing of several cytokines involving TNF-α, TGF-β and ILs [18]. The above complex inflammation circumstances result in hepatocytes apoptosis, collagen deposition, and abnormal proliferation of surviving liver cells, which would further result in hepatitis, liver cirrhosis and eventually liver cancer [19]. Although both clinical and experimental studies support the correlation between dyslipidemia and liver disease, there haven’t any study explored whether there has a specific lipid indicator could screen or predict LFTs abnormalities. Compared with indicators such as Non-HDL-C and HDL-C, Non-HDL/HDL has more advantages. As reported, Non-HDL usually includes VLDL, IDL, LDL and lipoprotein(a), which are usually called “bad cholesterol”, while HDL is called “good cholesterol”. Liver can synthesize and secrete these “bad cholesterol” to forward or “good cholesterol” to reverse deliver lipid from liver to periphery, so the level of Non-HDL/HDL in peripheral blood can cover more comprehensive abilities of liver metabolism than non-HDL or HDL alone. Herein, our research will explore whether this ratio can predict the occurrence of LFTs abnormalities.

Dividing the baseline data (year 2015) of 1745 participants into tertiles according to NonHDLc/HDLc ratio, we found proatherogenic variables such as BMI, serum lipid level, blood pressure and fasting glucose were worse in the highest tertile than the lowest one. As expected, ALT increased as the tertiles of the NonHDLc/HDLc ratio increased, and AST and TBil were not increased or decreased as the tertile increased (Table 1). We then calculated the three enzyme levels at the end of follow-up (year 2016) and found that AST and Tbil almost had no difference in three Tertiles and ALT persisted significant as the tertile increased (Table2). Collectively, these data illuminated the opinion that people with higher NonHDL/HDL ratio are more inclined to have higher ALT, and this ratio could significantly increase AST and minor increase TBil.

Both in univariate and multivariable linear regression models, the NonHDLc/HDLc ratio were significantly associate with LFTs abnormalities (newly occurrence abnormal ALT and AST). Similarly significant trends were also found in the situation of new occurrence of abnormal ALT but not AST (Table 3).

Serum aminotransferases such as ALT and aspartate AST indicate the enzymes leaked into circulation through damaged cytomembrane, and they are two most reliable markers of liver function [20–22]. ALT is present mainly in the cytosol of the liver and in low concentrations elsewhere; AST has cytosolic and mitochondrial forms and is present in tissues of the liver, heart, skeletal muscle, kidneys, brain, pancreas, and lungs, and in white and red blood cells [23]. Moreover, ALT is a marker of non-alcoholic fatty liver disease (NAFLD), owing to it could reflect inflammation and fatty change in liver [24, 25]. In the diagnosis of NAFLD, scholars even support that ALT could sometimes instead of imaging or invasive biopsies, with the latter being regarded as gold standard [25]. Besides, ALT may also be a good indicator of obesity, metabolic syndrome and cardiovascular disease which all associated with liver disease or LFTs abnormalities [26, 27]. In comparison, AST and total bilirubin failed to have the equally significant role in fatty liver disease or LFTs abnormalities, which are in consistent with our results.

In stratified analysis, the risk of LFTs abnormalities increased with a greater NonHDLc/HDLc ratio for female participants, BMI > 24 participants (Table 4). Postmenopausal women have a higher risk of obesity, and the latter is a important cause of liver disease as researches have reported [28, 29]. Due to the number of diabetic patients is relatively small and there may be a certain bias in the data, we failed to derive the relationship between NonHDLc/HDLc ratio and LFTs in them.

NAFLD is the most common form of chronic liver disease in the Western countries and increasingly important in other parts of the world [30]. In this study, we calculated the portion of LFTs abnormalities accompanied with NAFLD at the end of follow-up and found it increased higher as tertile increased (Additional file 1: Figure S1). Similarly, the portion of NAFLD also increased significantly as tertile increased (Additional file 2: Figure S2). These two figures showed the relationship between NonHDLc/HDLc and NAFLD and supported the ratio could predict incidence of LFTs abnormalities from another profile.

At last, we would like to explain why we choose elderly rather than young population in our research. Firstly, there exist more confounding and uncontrolled factors in the study of younger population. They are prone to overwork, stay up late and irregular diet (such as not have breakfast) plus the love of eating greasy, fried and spicy food. These behaviors will cause the decline of immunity, metabolic disorders, increased liver burden and eventually lead to LFTs abnormalities, thus, our current research focus on elderly population.

## Conclusion

In this large cohort of participants with normal LFTs at baseline, we demonstrated nonHDLc/HDLc ratio is an independent risk factor for LFTs abnormalities in geriatric population. Of course, further research may need to replicate these findings in other cohorts and elucidate the precise mechanisms underlying this association in experimental and clinical studies.

## Additional files

Additional file 1: **Figure S1.** The portion of chronic LFTs abnormalities and NAFLD patients according to Non-HDL/ HDL levels tertiles. The cut of values of Non-HDL / HDL ratio were < =2.23, 2.23–2.96, and > =2.96.#*P* value compared with Tertile 1,#*P* < 0.05. (TIF 1338 kb)

Additional file 2: **Figure S2.** The portion of NAFLD patients according to Non-HDL/ HDL levels tertiles. The cut of values of Non-HDL / HDL ratio were < =2.23, 2.23–2.96, and > =2.96.#*P* value compared with Tertile 1,#*P* < 0.05. ※ *P* value compared with Tertile 2, ※ *P* < 0.05. (TIF 2021 kb)

## Abbreviations

BMI: Body Mass Index
HDL-C: High Density Lipid Cholesterol
LDL-C: Low Density Lipid Cholesterol
LFTs: Liver function tests
non-HDL-C: non- High Density Lipoproteins Cholesterol
Tbil: Total bilirubin
