# Supplementary figures and images for: Non-HDL-cholesterol to HDL-cholesterol ratio is an independent risk factor for liver function tests abnormalities in geriatric population
Source: Lipids Health Dis. 2018 Dec 28;17:296. doi: 10.1186/s12944-018-0940-0 (PMC6311027; doi:10.1186/s12944-018-0940-0)

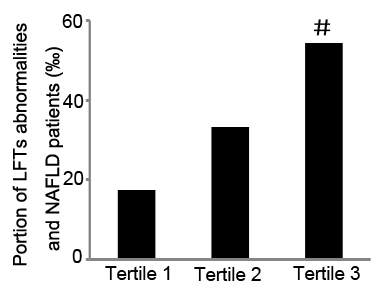

Supplement: Supplementary file 1 — Figure S1. The portion of chronic LFTs abnormalities and NAFLD patients according to Non-HDL/ HDL levels tertiles. The cut of values of Non-HDL / HDL ratio were < =2.23, 2.23–2.96, and > =2.96.#P value compared with Tertile 1,#P < 0.05. (TIF 1338 kb) [file 12944_2018_940_MOESM1_ESM.tif]

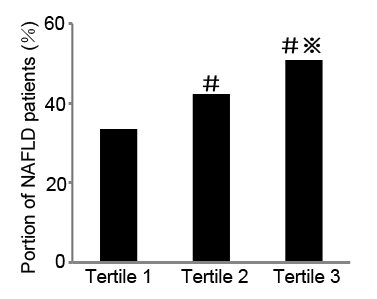

Supplement: Supplementary file 2 — Figure S2. The portion of NAFLD patients according to Non-HDL/ HDL levels tertiles. The cut of values of Non-HDL / HDL ratio were < =2.23, 2.23–2.96, and > =2.96.#P value compared with Tertile 1,#P < 0.05. ※ P value compared with Tertile 2, ※ P < 0.05. (TIF 2021 kb) [file 12944_2018_940_MOESM2_ESM.tif]
